# Management of an Extrusive Luxation Concomitant with Subluxation: A Case Report with Ten-Year Follow-Up

**DOI:** 10.1155/2021/6256894

**Published:** 2021-07-24

**Authors:** Saeedeh Mokhtari, Sepideh Hosseini, Maryam Khosrozadeh

**Affiliations:** Department of Pediatric Dentistry, School of Dentistry, Tehran University of Medical Sciences, Tehran, Iran

## Abstract

Traumatic dental injuries (TDIs) are a public health concern with high prevalence and incidence rates. Proper intervention can significantly reduce the subsequent complications of these events. This case report describes the clinical interventions to manage a patient with traumatized maxillary incisor with severe extrusive luxation. The procedure was aimed at preserving pulp vitality and providing periodontal maintenance to the highest possible degree. Owing to timely and accurate treatments, ten years of success have been achieved in this patient, as discussed in this case report.

## 1. Introduction

Traumatic injuries to permanent teeth are an ever-increasing problem due to traffic accidents, sports, physical activities, and violence, making them an integral part of the general dental practice [1]. In 18-33% of cases, pulpal/periodontal injuries ensue [2].

Extrusive luxation is a type of traumatic injury mainly caused by forces with oblique direction, characterized by the partial or total separation of periodontal attachments. Following injury, the loosening and axial displacement of the tooth are expected. This elongated tooth, also called “partial avulsion,” is threatened by loss of vascular supply and pulp vitality. The patient complains of pain during mastication; however, spontaneous discomfort is rare. Radiographically, an empty space around the apices of the extruded tooth is seen [3–5].

Subluxation is defined as an injury to the tooth and supporting tissues that results in abnormal and increased tooth mobility, but no displacement occurs [6–8].

Pulp tissue degeneration, pulpal calcification, and root resorption are among the most frequently reported complications after subluxation and luxation injuries. The root development stage, the oral bacterial load, and the severity of the injury are determinants of the final outcome of the treatment. Accordingly, pulpal and periodontal complications are highly incident in teeth with closed apices and can occur from weeks to years after the trauma [7, 9].

The psychological effects of traumatic dental injuries on parents and the child are a real issue. Children with untreated damaged teeth tend to have lower satisfaction with their appearance and exhibit low self-esteem, leading to some serious social problems [10, 11]. This case report focuses on treatment procedures, rationale, and ten-year follow-up of a patient with severe extrusive luxation and subluxation at the same time.

## 2. Case Presentation

**Figure 1:**
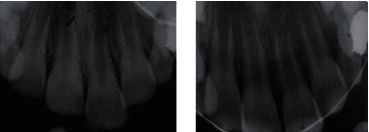
(a) Initial periapical radiograph. (b) Radiographic examination after *repositioning* is completed.

A healthy 7-year-old boy was referred to a pediatric dental clinic following a fall at school 3 hours after the accident. Extraoral examinations revealed soft tissue injuries, including upper lip abrasion and swelling. In addition, during the intraoral examination, severely extruded tooth #21 (about 4 mm), subluxation of teeth #11, #12, #22, #53, and #63, and laceration of the upper lip mucosa (about 3 cm) were observed. Radiographic assessment (XGenus, De Götzen SRL, Varese, Italy) confirmed the extrusive luxation of tooth #21, possible alveolar fracture, and incomplete root development of all the maxillary incisors (Figure 1(a)).

At the emergency appointment, the left central incisor was repositioned, and a wire-resin splint (0.7 mm in diameter, Dentaurum, Ispringen, Germany) was applied on maxillary central and lateral incisors, primary canines, and primary first molars (Figure 1(b)).

Radiographic evaluation of the soft tissue was also performed to detect any foreign body remnants in the upper lip. Then, the lip wound was sutured (*Coated VICRYL*® (*polyglactin 910*) *Sutures*, ETHICON Inc., USA). Antibiotic (amoxicillin suspension 250 mg/5 mL, 5 mL every 8 hours) and 0.2% chlorhexidine mouthwash (twice a day) were prescribed for 7 days. Adequate oral hygiene and soft food diet were recommended to the patient and his parents. At the first follow-up after one week, soft tissues showed satisfactory healing, and on the radiographic examination, an improvement was observed. Nevertheless, none of the teeth responded to pulp vitality tests.

**Figure 2:**
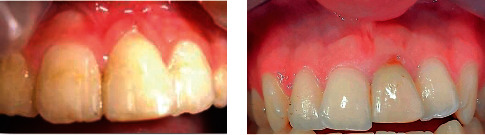
(a) The clinical view after the splint was removed and incisal adjustment was performed. (b) Clinical photograph at 10-year follow-up.

The splint was removed after four weeks according to the guidelines for the management of extrusive luxation [8, 12], and the incisal adjustment was performed (Figure 2(a)). However, the abnormal mobility of the left central incisor was recorded (grade III; even with vertical mobility). Thereby, central incisors and left lateral incisors were splinted again with the resin-bonding technique. Considering the immaturity and vulnerability of tooth #21 and after consulting the Department of Endodontics, we decided not to perform any endodontic intervention; instead, we monitored the pulpal condition closely. To ensure optimal healing of periodontal structures and any possible alveolar fractures, a longer splinting duration was considered.

At the follow-up appointment three weeks after injury, all the teeth responded to the pulp vitality test except for the maxillary left central incisor.

**Figure 3:**
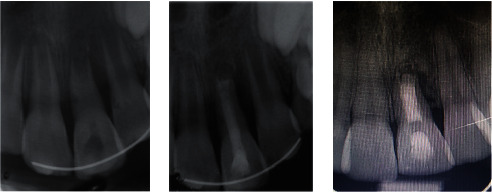
(a) Periapical radiograph showing the initiation of external root resorption on the left central maxillary incisor after 6 weeks. (b) Placement of an MTA plug and root canal obturation with gutta-percha after the resorption process ceased 9 months later. (c) The same tooth at 10-year follow-up.

The third follow-up after 6 weeks revealed external root resorption and vertical bone loss on radiographic examination of tooth #21 (Figure 3(a)); therefore, endodontic treatment was undertaken. Working length was verified by an apex locator (Woodpecker, Foshan, China) and radiography. The root canal was lightly instrumented using K-files, followed by copious irrigation with 2.5% sodium hypochlorite and saline solutions. Then, the root canal was dried with endodontic paper points (Dentplus, Choonchong, Korea). The mixture of calcium hydroxide (Golchadent Co., Karaj, Iran) and saline was placed into the root canal. The access cavity was sealed with RMGI (Fuji II LC, GC; Tokyo, Japan). Calcium hydroxide was replaced regularly every 3 months. After 9 months, the resorption process stopped. Calcium hydroxide was rinsed out by instrumentation and irrigation with 2.5% NaOCl and saline solutions. MTA (MTA-Ang; Angelus, Londrina, PR, Brazil) was mixed according to the manufacturer's instructions and placed in the apical one-third of the root canal using an MTA carrier until a thickness of 4-5 mm was achieved. The correct position and thickness of the MTA plug were checked with a periapical radiograph. A moist cotton pellet was placed in the root canal to ensure the proper setting of MTA, and then, the access cavity was sealed with RMGI. After 3 days, the rest of the root canal was obturated with gutta-percha (Meta Biomed Co., Korea) and sealer (Dentsply/Maillefer, Tulsa, OK) (Figure 3(b)). The access cavity was restored with composite resin (Clearfil AP-X (Kuraray Medical, Tokyo, Japan)). The patient was then arranged for 1-, 3-, 6-, and 12-month follow-ups to monitor the healing of the radiolucent lesion adjacent to the left central incisor.

At a 10-year recall, the teeth were asymptomatic. Despite some minor radiolucency caused by a subtle vertical bone defect detectable along the distal portion of the root, clinical and radiographic features of the left maxillary central incisor seemed stable (Figures 2(c) and 3(c)), and normal mobility and percussion sound were recorded; a modest degree of infraocclusion was also evident.

From the periodontal point of view, no attachment loss was observed, and the probing depth was 1.5 mm in the treatment area.

## 3. Discussion

Dental traumatic injuries (TDIs) are more common in school and preschool children, accounting for 5% of all accidental injuries. Immature teeth and pubertal growth spurt complicate the treatment of these cases. As a result, there is a worldwide controversy in the management of TDIs. The key to ensure a successful outcome is an accurate diagnosis, treatment plan, and regularly arranged monitoring appointments [8].

Teeth are usually affected by a combination of several injuries. The main purpose of the existing guidelines is to improve the management of injured teeth and minimize trauma-related complications. The maintenance of the periodontium must be the first priority in the management of luxation injuries. In addition, soft tissue injuries and repositioning should be considered before endodontic procedures.

Short-term, passive, and flexible splints for splinting luxated, avulsed, and root-fractured teeth are recommended to allow physiological movements. To ensure maximum healing and avoid plaque retention, composite resin and bonding agents should be kept away from the gingival tissues and proximal areas. There is limited evidence to support the use of antibiotics, mainly at the clinician's discretion in the case of soft tissue and other associated injuries [8, 13].

A variety of complications, such as pulp necrosis, pulp canal obliteration, root resorption, and loss of marginal attachment, might occur after trauma [14]. The degree of displacement, treatment delay, root maturation, and concomitant crown and root fractures are among the determinative factors in the prognosis of luxated teeth [1].

Pulp vitality assessment is a key diagnostic procedure in endodontics. Since it is not feasible to directly examine the pulp tissue, indirect methods are suggested to record pulpal conditions [15]. The diagnosis of pulp necrosis in immature teeth would be confusing. Because of the limitations related to radiological analysis, evaluation of the clinical signs and symptoms, such as the presence of pain, tenderness to percussion, abnormal mobility, crown discoloration, or a sinus tract, seems to be more reliable. Radiographic comparison of the root formation of the tooth in question with the contralateral tooth helps assess whether the root growth for that patient occurs at the normal rate [1].

The risk of inflammatory resorption should be weighed against the likelihood of pulp recovery and revascularization. This type of resorption occurs progressively and aggressively in children. This emphasizes the importance of regular follow-ups. In the discussed case, the inflammatory resorption observed was highly aggressive. Due to the incomplete development of the root and the crown-root ratio, if it was not halted, the continuation of this resorption could lead to the definite loss of the tooth.

Apexification or pulp space revascularization/revitalization procedures are the techniques used in the endodontic management of necrotic teeth with incompletely formed roots [8]. Given that replacement resorption results from crushing rather than tearing injuries, it seems to be a rare phenomenon in extrusive accidents [16]. However, in this case, there was an apparent infraocclusion in the left central incisor and a bone defect showing incomplete healing of the bone in the area. These can indicate areas of minor ankylosis affecting the root of the mentioned tooth.

After 10 years of follow-up, no adverse sequelae or dental complications were found in this case. Despite the residual bone lesion and some degree of ankylosis, the tooth is clinically asymptomatic and functional. Therefore, this can be considered a successful treatment in a long-term follow-up. The success of the procedure is determined by the consistency of the dentist-patient relationship and the proper execution of protocols in various cases of dental injury.

## 4. Conclusion

The present case report shows a favorable and stable outcome of timely treatment with a ten-year follow-up. However, considering the high prevalence of traumatic events and the significant impact of anterior teeth on self-confidence and quality of life, success in the treatment of these accidents necessitates professional knowledge and experience, accuracy, and regular follow-up sessions.
